# Knowledge and attitudes on oral health of women during pregnancy and their children: an online survey

**DOI:** 10.1186/s12903-023-03732-2

**Published:** 2024-01-16

**Authors:** Maria Grazia Cagetti, Claudia Salerno, Andrei Cristian Ionescu, Serena La Rocca, Nicole Camoni, Silvia Cirio, Guglielmo Campus

**Affiliations:** 1 Department of Biomedical, Surgical and Dental Sciences, University of Milan, Via Beldiletto 1, 20142 Milan, Milan Italy; 2 Department of Restorative, Preventive and Pediatric Dentistry, University of Bern, Freiburgstrasse 7, 3012 Bern, Switzerland; 3 Department of Biomedical, Surgical and Dental Sciences, University of Milan, Via Pascal 36, 20133 Milan, Milan Italy; 4 Department of Surgery, Microsurgery and Medicine Sciences – School of Dentistry University of Sassari, Sassari, Italy. Viale San Pietro, 43. Department of Restorative, Preventive and Pediatric Dentistry, University of Bern, Freiburgstrasse 7, 3012 Bern, Switzerland

**Keywords:** Oral health, Pregnancy, Children, New mothers, Questionnaire, Knowledge, Behavioural, Survey

## Abstract

**Background:**

Life-long healthy behaviors are established during pregnancy and the first years of life. In this cross-sectional survey, new mothers with a high level of schooling living in Northern Italy (Lombardy Region) were interviewed to assess their knowledge and attitudes towards their and child oral health.

**Methods:**

A questionnaire (27 items) was developed to assess socio-demographic factors, knowledge, and attitudes towards maternal and child oral health. The questionnaire was disseminated in perinatal courses, private gynecological clinics, and *via* social media. Mothers aged ≥18 years, with at least a child aged 0–36 months, with a high school diploma or higher, were included in the survey.

**Results:**

A total of 1340 women completed the questionnaire, 1297 of whom had a child aged 0–36 months, 792 lived in Lombardy, and 600 had a high level of education and were finally included. About half of the sample (44.67%) was aged between 31 and 35 years, 76.50% were employed, and the majority had only one child (81.50%). During pregnancy, 28.33% of the sample reported problems with teeth and gums, while only 36.00% visited a dentist. More than 40% of the sample said they were not aware of a possible link between oral health and pregnancy, and 73.17% had not received any advice about their oral health or the future health of their baby’s mouth. Less than 20% of women were aware of the increased caries risk associated with prolonged or night-time breastfeeding. Better knowledge/attitude was associated with the age of the child (*p* < 0.05), the number of children (*p* < 0.05) and whether the mother had received advice during pregnancy (*p* < 0.05).

**Conclusions:**

The results of this survey show a lack of dental care during pregnancy, a lack of information about oral health from health professionals during and after pregnancy, and consequently gaps in the knowledge needed to care for the oral health of the woman and her child. There is a need for training in oral health for pregnant women and new mothers, but also a need for behavioural change among health professionals who care for pregnant women.

**Supplementary Information:**

The online version contains supplementary material available at 10.1186/s12903-023-03732-2.

## Background

Pregnancy and postpartum are adequate periods for adopting a healthy lifestyle, as motivation to follow new rules is higher than at other times [1, 2]. Good practices include regular exercise [1], and a balanced diet to control weight gain [3, 4]. Then again, alcohol, drugs, and smoking are known as dangerous habits for mothers and their offspring [5, 6]. Midwives and maternal care professionals provide educational and preventive interventions for maintaining a healthy lifestyle in pregnant and postpartum women [7]. Such interventions have positive effects by minimizing the use of medical interventions improving the health of both mother and child [8, 9]. However, while the above lifestyle concepts are well disseminated, others, such as oral health during pregnancy and in the perinatal period, are not often considered by maternal health professionals and, as a result, pregnant women are not sufficiently aware of them [10].. Obstetricians and gynecologists should receive up-to-date dental education to minimize the adverse outcomes of poor oral health in the prenatal and postnatal [11, 12].

Dental research emphasizes that the first thousand days of life are crucial for implementing actions to ensure good oral and general health development that can last a lifetime [13]. Clinical evidence of poor oral health in the perinatal period can be summed up as follows:

- there is an association between the risk of preterm delivery and low birth weight with periodontal disease and increased oral inflammatory burden in pregnant women [14, 15]. Periodontal disease can be controlled with regular dental check-ups and proper oral hygiene at home, using a toothbrush and dental floss at least twice daily;
- the overall prevalence of oral mucosal disorders is 11.8% in pregnant women. Gingival hyperplasia, *morsicatio buccarum*, oral candidiasis, pyogenic granuloma, and benign migratory glossitis are the most common lesions [16]. Regular check-ups can reduce their occurrence or manage them appropriately, reducing related pain;
- reflux, which is very common during the first trimester, can lead to dental erosion, so appropriate preventive measures should also be recommended [17];
- mothers with a high salivary level of *mutans* streptococci, a major caries-related bacteria, can favor early colonization of the child’s mouth, increasing baby caries risk [18, 19]. Therefore, salivary bacteria transmission between the mother’s and baby’s mouths should be discouraged;
- breastfeeding beyond 12 months, especially at night, is positively associated with Early Childhood Caries (ECC) [20]. Providing advice on diet and feeding leads to a reduced risk of caries in children [21]. When the first tooth erupts, it should be brushed with fluoride-containing toothpaste.

Caregivers, particularly mothers, play a crucial role in their children’s oral health, not only because of individual and biological factors but also because their socio-economical determinants are related to infants’ caries risk [22]. The experience of ECC and dental pain in children is related to parents’ educational and economic level: the most disadvantaged mothers have offspring with the highest prevalence of dental caries in both dentitions [23]. In a study conducted in the USA, the cumulative risk of caries at three years was lower among children whose mothers had more than a high school diploma [24]. Similar conclusions can, however, be found in studies held in high [25, 26], middle [27], and low-income countries [28, 29]. Good oral health behavior, including early referral of children to dental services, is associated with higher maternal education and oral health knowledge [27]. For these reasons, pregnant women and their partners should be educated about the benefits of good oral health practices. Parents can only help their children establish effective dental hygiene and a proper eating routine if they know the harmful effects of incorrect behavior.

Based on these premises, a cross-sectional web-based survey was conducted in Northern Italy, interviewing new mothers with a high level of education to assess whether their knowledge and attitudes about their own and their child’s oral health were adequate.

## Methods

The study was designed as an observational, questionnaire-based, cross-sectional study; it complied with the Declaration of Helsinki and was performed according to ethics committee approval (Ethics Committee Board of Sassari University, Sassari, Italy, 05/24/2022 N° 275). The reporting of this study follows the Standards for Reporting of Diagnostic Accuracy guidelines [30].

The questionnaire was developed based on two previously validated questionnaires [31–33] and consisted of three main sections (Supplementary file S1):

- section I: Demographic characteristics, including mothers’ age, employment, educational level, and so on (7 items);
- section II: Mothers’ oral health knowledge and attitudes during pregnancy (7 items);
- section III: Children’s oral healthcare knowledge and attitudes reported by their mothers (12 items).

In section III, a system was developed to score the items based on the number of correct/favorable answers given by responders: correct/favorable answers were scored as 1, and other answers as 0 (min 0; max 12).

The questionnaire was translated from English into Italian by two native Italian-speaking translators fluent in English and experienced in the topic. After the translation, a consensus version was identified; then it was retro-translated into English by a third person not involved in the study to ensure the accuracy and comparability of the translation. A quantitative analysis of the accuracy of the questionnaire was performed by submitting it to 10 experts (4 dentists specialized in Paediatric Dentistry with more than 5 years of experience, 3 academics, and 3 clinical researchers). The quantitative content validity of each item was assessed using the Content Validity Index (CVI) and the Content Validity Ratio (CVR) [34]. The Scale Content Validity Index (S-CVI) was finally calculated using the universal agreement method. The CVI and S-CVR for the entire tool were both 0.98 based on experts’ opinions (Supplementary file S2).

The final Italian version was pre-tested in March 2022 for comprehensibility on a small sample of 17 women not included in the survey. After completing the questionnaire, they were contacted to find out if they had experienced any difficulty in understanding the questions and were given a comprehension score from 1 (extreme difficulty) to 5 (no difficulty). A result of 4.54 ± 0.17 was obtained. Participants in this pilot test made some suggestions about the wording, and the questionnaire was modified accordingly.

An online version of the anonymous questionnaire was created using Google Form (Google LLC, Mountain View, CA, USA) and made accessible via a QR code. A convenience sample of leisure and gynecological centers was selected by an online search and contacted by phone. In order to try to select mothers with a high educational and socioeconomic level, leisure centers (mainly gyms and swimming pools) where private antenatal and postnatal courses are held (mainly Yoga and aqua gym) and private gynecological practices in Milan, Northern Italy were chosen. In those available, the flyer with the QR code was distributed. In addition, the QR code was shared on the Instagram pages of some communities dedicated to perinatal care. A description of the purpose of the study was also inserted before the first question, and mothers were asked to sign an online informed consent under Italian data protection laws. If they did not sign the consent, the questionnaire was automatically closed.

The following inclusion criteria were used for enrolment:

- signing the online informed consent;
- be 18 years of age or older;
- have at least one child aged between 0 and 3 years;
- be a resident of Northern Italy (Lombardy Region);
- hold a high school diploma or higher educational degree.

According to the National Institute of Statistics, there were 148,884 children between 0 and 3 years of age in Lombardy in 2022, with almost the same number of new mothers [35]. The sample size was calculated based on data in the literature, considering the possibility of non-responders. From the two validated questionnaires mentioned above [31, 32], two questions were used to calculate the sample size: one on mothers’ oral health during pregnancy and the second on children’s oral health. Both variables were calculated at _95%_CI with a significance level of 0.05. The highest result of the two calculations, equal to 377 mothers, was then used for the study. Data were collected between June and December 2022.

### Statistical analysis

All data obtained from the included questionnaires were inserted in a spreadsheet (Microsoft™ Excel™ 2019 for Mac) for statistical processing and analysis. Descriptive statistics for all variables were performed using frequencies (N/%). Normality and heteroskedasticity of data were assessed with Shapiro-Wilk’s and Levene’s tests (α = 0.05). The association between the items’ answers of Section II of the questionnaire was evaluated using the Chi-square or Fisher’s exact test according to data distribution. The relation between the median and IQR of the Total score and the socio-demographic characteristics as mother’s age (≤ 35 vs > 35 years), children’s age (0–6 months vs > 6 months), number of children (1 vs > 1), and receiving information during pregnancy (yes vs no) was assessed with the Mann-Whitney U test. The correlation between the median and IQR of the Total score and from whom mothers received information was assessed with the Kruskal-Wallis test. If the null hypothesis of Kruskal-Wallis’s test was rejected, post-hoc pairwise analyses were performed with Dunn-Bonferoni’s test. The alpha risk was set to 5% (α = 0.05).

## Results

The questionnaire was opened by 1340 subjects, 1297 of whom had a child aged 0–3 years, 792 were residents in Lombardy, and 600 had a high level of education (high school diploma or higher), so the latter respondents (*n* = 600) were included and analyzed. About half of the sample (44.67%) were aged between 31 and 35 years, 76.50% were employed, and most had only one child (81.50%) aged between 0 and 6 months (55.33%) (Table 1).

**Table 1:** Section I: demographic characteristics of respondents

Variables	N	%
Age group		
< 25 years	8	1.33
25–30 years	70	11.67
31–35 years	268	44.67
36–40 years	189	31.50
> 40 years	65	10.83
Nationality		
Italian	576	96.00
Other	24	4.00
Employment status		
employee	459	76.50
self-employed	115	19.17
housewife	6	1.00
occasional job	4	0.67
unemployed	16	2.66
N of children		
One	489	81.50
Two	106	17.67
Three	3	0.50
More than three	2	0.33
Last child age		
0–6 months	332	55.33
7–12 months	133	22.17
13–24 months	71	11.83
25–36 months	64	10.67

Table 2 describes mothers’ knowledge and attitudes toward oral health during pregnancy.

**Table 2:** Section II: Mothers’ oral health knowledge and attitudes during pregnancy

Items	Yes	No
	N (%)	N (%)
Did you experience any problems with your teeth or gums during your pregnancy?	170 (28.33)	430 (71.67)
Did you regularly visit the dentist/dental hygienist before your pregnancy?	443 (73.83)	157 (26.17)
Did you visit the dentist/dental hygienist during pregnancy?	216 (36.00)	384 (64.00)
If the answer is ‘no’, give one closest reason why you did not go to the dentist.		
Because I thought my gums would soon recover	26 (7.77)	
Because I knew that mouth problems can be pregnancy-related	12 (3.13)	
Because the dentist could have used drugs without consulting my gynecologist	10 (2.60)	
Because the dentist/dental hygienist could not have done any treatment	23 (5.99)	
Because I had no oral problems during pregnancy	245 (63.80)	
No specific reason	68 (17.71)	
Other reason	0 (0.00)	
Do you think that gum problems, such as bleeding when brushing teeth, can influence the course of pregnancy and/or the health of the baby at birth?	125 (20.83)	475 (79.17)
Have you ever heard of a possible relationship between oral health and pregnancy?	352 (58.67)	248 (41.33)
If the answer is ‘yes’, where did you hear this? (*More than one answer was possible*)		
I read it in a book/journal/online resources	147	
My gynecologist told me about it	45	
My dentist/dental hygienist told me about it	111	
I heard it from friends/colleagues/relatives	106	
I experienced this during pregnancy	45	
Did you receive advice on your oral health during pregnancy or on the future health of your child?	161 (26.83)	439 (73.17)
During pregnancy, who give you advice on your oral health or that future of the baby?		
General practitioner	1 (0.62)	
Gynecologist	23 (14.29)	
Obstetrician	3 (1.86)	
Dentist/dental hygienist	121 (75.16)	
Other	13 (8.07)	

Only 28.33% of mothers reported that they had experienced oral health problems during pregnancy. Most (73.83%) visited the dentist regularly before pregnancy, but only 36.00% visited the dentist during pregnancy. Among the reasons for limited visits, the most common was the lack of oral problems (63.80%). Furthermore, only 20.83% of the mothers knew that periodontal problems could affect pregnancy outcomes, and 41.33% had never heard of a possible link between oral health problems and pregnancy. Of the mothers who stated that they were aware of a possible link between oral health and pregnancy, only 156 reported that they had learned about it from their gynecologist or dentist/dental hygienist. However, 73.17% of the mothers reported never receiving specific instructions about personal or child oral health during pregnancy. Of the 161 (26.83% of the total sample) mothers who received oral health advice, the majority (75.16%) got it from their dentist/dental hygienist.

The yes/no response questions of section II of the questionnaire were also analyzed by stratifying the sample into two subgroups: mothers who had gone to the dentist/dental hygienist during pregnancy and mothers who had not (Table 3). All the analyzed variables differed significantly between the subgroups (*p* < 0.05 for all). A higher percentage of mothers who experienced oral problems was detected among the ones who visited the dentist (91 vs 79), even if the majority in both subgroups did not experience oral problems. A higher knowledge of oral health and pregnancy was found among the mothers who visited the dentist during their pregnancy compared to the ones who did not. Of the mothers who claimed to have visited the dentist during pregnancy and received information on oral health, 87.12% were informed by this health professional (data not in table).

**Table 3:** Oral health problems, dental care, and mother’s knowledge stratified by dental visits during pregnancy (yes/no)

Items	Answers	Dental visit during pregnancy		*p*-value
		Yes = 216	No = 384	
		N (%)	N (%)	
Did you experience any problems with your teeth or gums during your pregnancy?	Yes	91 (42.13)	79 (20.57)	< 0.05
	No	125 (57.87)	305 (79.43)	
Did you regularly visit the dentist/dental hygienist before your pregnancy?	Yes	197 (91.20)	246 (64.06)	< 0.05
	No	19 (8.80)	138 (35.94)	
Do you think gum problems, such as bleeding when brushing teeth, can influence the course of pregnancy and/or the health of the baby at birth?	Yes	74 (34.26)	51 (13.28)	< 0.05
	No	142 (65.74)	333 (86.72)	
Have you ever heard of a possible relationship between oral health and pregnancy?	Yes	157 (72.69)	195 (50.78)	< 0.05
	No	59 (27.31)	189 (49.22)	
Did you receive advice on your oral health during pregnancy or on the future health of your child?	Yes	132 (61.11)	29 (7.55)	< 0.05
	No	84 (38.89)	355 (92.45)	

**Fig. 1:**
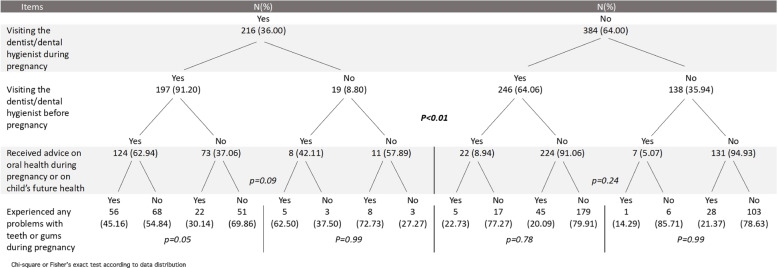
Cascade analysis system starting by dividing the sample between mothers who did or did not visit the dentist during pregnancy

A cascade analysis system was developed, starting by dividing the sample between mothers who did or did not visit the dentist during pregnancy, followed by a stratification based on who visited the dentist before pregnancy (*p* < 0.05), then according to having received oral health advice during pregnancy and finally according to the experience of oral problems during pregnancy. No significant differences were found in the last two stratifications (*p*-values ranged from 0.05 to 0.99) (Fig. 1).

Regarding items from section III, based on the scoring criteria described above, the mean knowledge score was 3.95 ± 1.14, that of attitude was 4.45 ± 1.13, and the Total score was 8.39 ± 1.85 (Table 4). Overall, 85.17% of the sample obtained more than half of the total score (max score = 12), while concerning the knowledge score (max score = 6), 88.17% obtained more than half of the maximum score. Regarding the attitude score, 84.33% obtained more than half of the maximum score (max score = 6, data not in table). The items for which the mothers were least prepared were the best option for treating a decayed primary tooth, the age at which it would be appropriate to make the first dental visit and the relationship between breastfeeding and caries.

**Table 4:** Section III: Knowledge and attitudes regarding children’s oral healthcare reported by mothers

Knowledge	Items	Desirable answer	Not desirable answer		Score
		N (%)	N (%)	N (%)	mean ± SD
		Yes	I don’t know	No/wrong	
	Primary teeth are important	548 (91.33)	51 (8.50)	1 (0.17)	0.91 ± 0.28
	Knowing what causes a cavity	522 (87.00)	35 (5.83)	43 (7.17)	0.87 ± 0.34
	Good oral health of the baby is linked to good general health	509 (84.83)	86 (14.33)	5 (0.84)	0.85 ± 0.36
	Multiple choice questions				Score
		Correct	I don’t know	Wrong	
	The role of fluoride in the toothpaste is to				
	Prevent tooth decay	431 (71.83)			
	Prevent gum problems			66 (11.00)	
	Give freshness			3 (0.50)	
		431 (71.83)	100 (16.67)	69 (11.50)	0.72 ± 0.45
	The best option for treating a carious primary tooth is				
	Extraction			64 (10.67)	
	Restoration	273 (45.50)			
	Drugs (Antibiotic/pain relief drug)			1 (0.17)	
		273 (45.50)	262 (43.67)	65 (10.83)	0.45 ± 0.50
	Age (years) when a child should receive the first dental visit				
	< 1 year	84 (14.00)			
	< 3 years			263 (43.83)	
	< 6 years			109 (18.17)	
	> 6 years			7 (1.17)	
	Only if problems occur			5 (0.83)	
		84 (14.00)	132 (22.00)	384 (64.00)	0.14 ± 0.35
	Overall Knowledge score				3.95 ± 1.14
Attitude		Agree	I don’t know	Disagree	Score
	A balanced diet is essential for a child’s growth and oral health	590 (98.33)	10 (1.67)	0 (0.00)	0.98 ± 0.13
	Bed-time bottle/breastfeeding can cause tooth decay	112 (18.67)	268 (44.67)	220 (36.66)	0.19 ± 0.39
	Frequent and prolonged bottle/breastfeeding can cause tooth decay	92 (15.33)	297 (49.50)	211 (35.17)	0.15 ± 0.36
	Child’s teeth should be brushed/cleaned since their first appearance	528 (88.00)	63 (10.50)	9 (1.50)	0.88 ± 0.32
	Tooth decay is caused by bacteria that are transmitted by sharing feeding utensils	211 (35.17)	222 (37.00)	167 (27.83)	0.35 ± 0.48
	I believe that using a pacifier dipped in honey, sugar or other substances can be harmful to a baby’s milk teeth	565 (94.17)	31 (5.17)	4 (0.66)	0.94 ± 0.23
	Overall Attitude score				4.45 ± 1.13
	Total score				8.39 ± 1.85

**Fig. 2:**
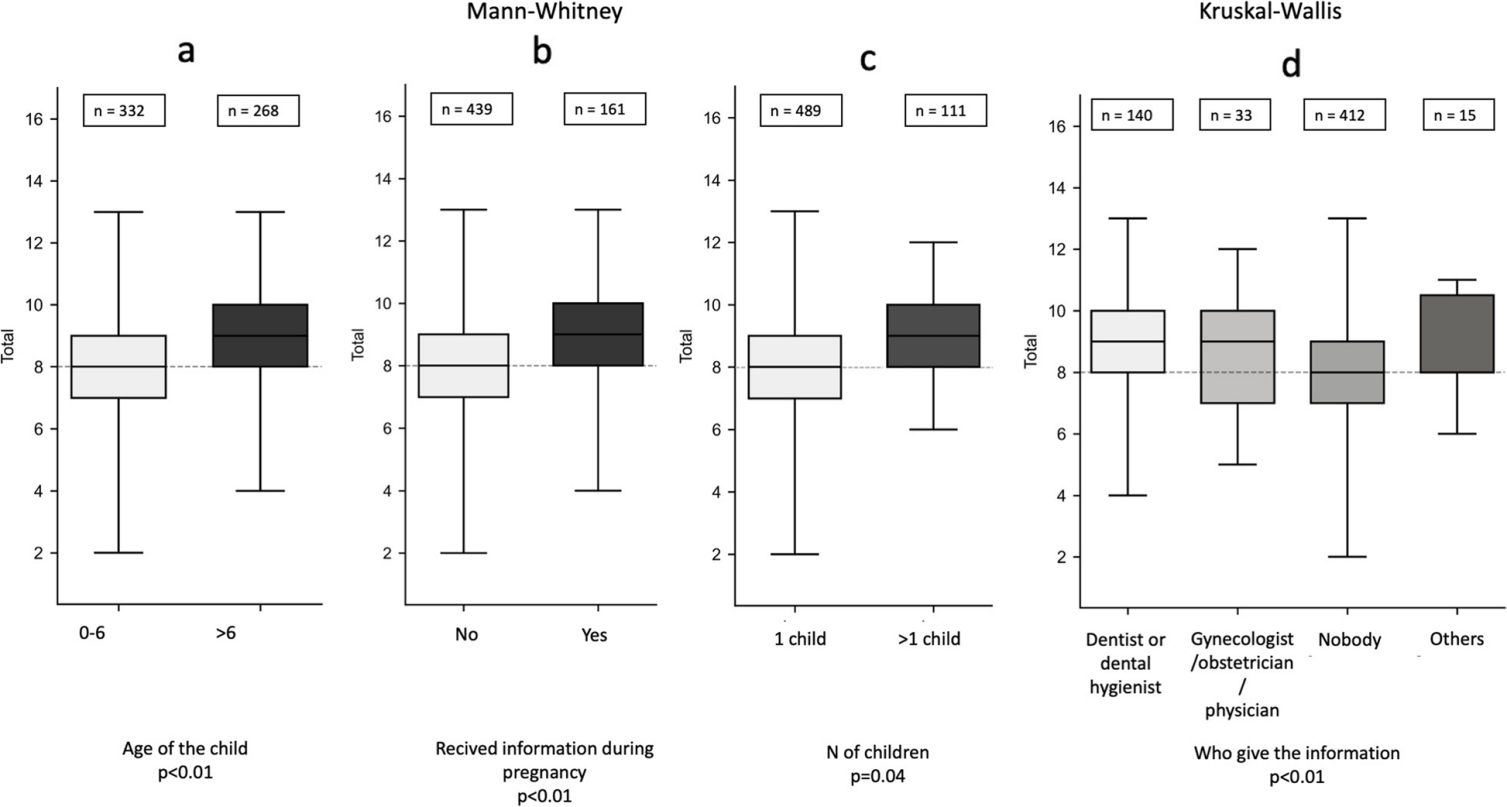
Association between Total score, age, number of children, and knowledge received

The achieved Total score was not associated with the mother’s age but with the age of the children, the number of children, whether the mother had received information during pregnancy, and from whom. The median score values were 8.0 (IQR 3.0) and 8.0 (IQR 2.0) in mothers younger/older than 35 years, respectively (*p* = 0.176) (not included in Fig. 2); 8.0 (IQR 2.0) and 9.0 (IQR 2.0) in mothers with children aged 0–6 months/more than 6 moths, respectively (Fig. 2a) (*p* < 0.05); 9.0 (IQR 2.0) and 8.0 (IQR 2.0) in mothers who received/did not receive information during pregnancy, respectively (Fig. 2b, *p* < 0.05); 9.0 (IQR 2.0) and 8.0 (IQR 2.0) in mothers with more than 1 child/only 1 child, respectively (Fig. 2c, *p* = 0.04) and finally, 9.0 (IQR 2.0), 8.0 (IQR 2.0), 9.0 (IQR 3.0) and 8.0 (IQR 2.5) in mothers who received information primarily by “dentist or dental hygienist”, “nobody”, “gynecologist or obstetrician or physician” and “others”, respectively (Fig. 2d) (*p* < 0.05). Pairwise analyses revealed differences for “nobody” *vs* “dentist or dental hygienist” (*p* < 0.05) and “nobody” *vs* “gynecologist or obstetrician or physician” (*p* = 0.05).

## Discussion

This survey aimed to assess whether the oral health knowledge and attitudes of new mothers with a high level of education were adequate to ensure a good level of oral health for themselves and their offspring. What emerged is a lack of dental care during pregnancy, a lack of information on oral health received from the various medical personnel during and after pregnancy, and, consequently, gaps in the knowledge needed to take care of her own and her child’s oral health. Such knowledge is mandatory to apply correct hygiene and dietary habits, such as rational use of fluoride and behaviors that reduce the risk of developing caries and periodontal problems [36]. Physical well-being derives from the organism’s health as a whole, including the connections between oral and general health; it is also intertwined with psychological and social well-being. Lack of knowledge leads to lower general and oral health [37]. The literature is replete with evidence of this relationship: mothers from lower socioeconomic backgrounds and with less education are more likely to have children with poorer health than mothers from higher socioeconomic backgrounds and with more education [38]. This connection is also present in high-income, developed countries like Italy. Recently, a high caries prevalence was retrieved in children with non-European backgrounds of the parents [39].

Correct hygiene habits are essential to keep the mouth healthy during this period of a woman’s life, as the oral cavity’s inflammatory processes can also negatively affect pregnancy outcomes [40]. A key finding of this survey is the low prevalence of women visiting a dentist or dental hygienist during pregnancy. Even those who visited the dentist regularly before pregnancy stopped doing so during pregnancy. This occurrence is probably due to a lack of information or a misconception that dental treatment during pregnancy can be dangerous or unnecessary. Gynecologists and midwives, as reported by the participants, seem unwilling to invest time and effort in their patients’ oral health. In agreement with these findings, a recent systematic review concluded that gynecologists, obstetricians, nurses, and general practitioners are aware of the importance of oral health during pregnancy but still fail to translate this knowledge into clinical practice [41]. A non-negligible percentage of women reported experiencing oral problems during pregnancy, in line with those reported in American and Canadian women samples [42, 43]. However, a large percentage of women who reported experiencing oral problems during pregnancy did not visit the dentist, especially if they had not been informed about it.

In contrast, a relationship between perceived need and dental visits during pregnancy has been reported in the literature. Women with reported symptoms of gingivitis or dental pain were more likely to seek dental services [44]. Therefore, dental and non-dental health professionals should pay more attention to health education in favor of oral health as well. Although the Regional Health Service in Lombardy offers pregnant women preventive dental and periodontal protocols at their request, free-of-charge preparatory courses during pregnancy do not cover oral health to date [45].

Less than half of the sample knew that primary teeth could be restored if decayed and believed the correct age for their child to have a dental visit was beyond three. Early examination, within the first year of life, has proven essential for preventing Early Childhood Caries, as it can intercept erroneous dietary and oral hygiene habits at an early stage [46]. In Italy, the prevalence of caries in preschool age is still high [47] and statistically significantly associated with socioeconomic inequality. This figure is unsurprising since knowledge and correct behavior still need to be fully acquired, even among the most educated mothers. It was speculated that not going to the dentist during pregnancy and the late recourse to a first visit for their child were more related to a lack of knowledge than to a lack of financial resources. Mothers with more than one child, although representing a minority of the sample, seem better informed on oral health topics, suggesting that experience played a more significant role than education received.

In Italy, oral health care is almost entirely provided by private practitioners; it is mainly financed by direct payment from patients or, to a lesser extent, covered by voluntary health insurance. In this situation, the family’s ability to spend is essential in access to dental care. The Public Health Service offers dental treatment up to age 14, but only some get treatment because of a minimal offer compared to the demand. For this reason, most children are not treated in public facilities and turn to private practitioners, even though there is a higher number of public dental clinics in Northern Italy compared to the rest of the country. These reasons reduce regular dental check-ups and treatments in low-income population groups.

Lombardy, located in Northern Italy, ranks first in terms of resident population and economic status [48]; it was considered the ideal place to enroll subjects with the considered requirements, that is, mothers with a high socioeconomic standard, with a high level of education and a job.

A strength of this survey, which aimed to explore mothers’ knowledge that they should be well informed and consequently behave correctly concerning oral health, is that it provides a broad overview of mothers’ knowledge about their and their children’s oral health. The lack of data on individual income could be a limitation, as income was not investigated to protect the participants’ privacy, even though the questionnaire was anonymous. The income information, when analyzed together with the level of education, could have added helpful information to better understand the mothers’ habits. Furthermore, the role of the pediatrician as a health professional who could have provided valuable indications, at least for the child’s health, needed to be explicitly investigated.

## Conclusions

The results of this survey show a lack of dental care during pregnancy, a lack of information on oral health received from medical personnel during and after pregnancy, and, consequently, gaps in the knowledge needed to care for one’s oral health and that of the child.

This situation highlights how essential it is to promote oral health training courses for pregnant women and new mothers. There is also a need to improve the knowledge and skills of health workers caring for pregnant women and young children to change their habits and promote oral prevention messages during their check-ups.

### Supplementary Information

**Additional file 1.**

## Data Availability

The datasets used and analyzed during the current study are available from the corresponding author upon reasonable request.

## Abbreviation

ECC: Early Childhood Caries
