# Supplementary material for: Knowledge and attitudes on oral health of women during pregnancy and their children: an online survey
Source: BMC Oral Health. 2024 Jan 16;24:85. doi: 10.1186/s12903-023-03732-2 (PMC10790411; doi:10.1186/s12903-023-03732-2)
Supplement: Supplementary file 1 — Additional file 1. [file 12903_2023_3732_MOESM1_ESM.docx]

Supplementary file

S1. Study questionnaire

S2. Content Validity Index and Content Validity Ratio

S1. Study questionnaire

**Part 1. Demographic data**

1. Please indicate which of the following age groups you fall into:
2. < 25 years of age
3. 25-30 years of age
4. 31-35 years of age
5. >40 years of age
6. Your nationality is:
   1. Italian
   2. other nationality
7. Your occupation is:
   1. unemployed
   2. housewife
   3. occasional job
   4. employee
   5. self-employed
8. How many children do you have?
   1. 1
   2. 2
   3. 3
   4. 4
   5. >4
9. How old is your younger child?
   1. 0-6 months
   2. 7-12 months
   3. 13-24 months
   4. 25-36 months

**Part 2. Oral health during pregnancy**

1. Did you experience any problems with your teeth or gums during pregnancy?
   1. Yes
   2. No
2. Did you regularly visit the dentist/dental hygienist before your pregnancy?
   1. Yes
   2. No
3. Did you visit the dentist/dental hygienist during pregnancy?
   1. Yes
   2. No
4. If the answer is ‘no’, give one closest reason on why you didn’t go to the dentist
   1. Because I thought my gums would soon recover
   2. Because I knew from my previous pregnancy that mouth problems can be pregnancy-related
   3. Because the dentist could have used a local anesthetic or prescribed me antibiotics without consulting my gynecologist
   4. Because the dentist/dental hygienist could not have done any treatment during pregnancy
   5. Because I had no oral problem during pregnancy
   6. No specific reason
   7. Other reason
5. Do you think that gum problems, such as bleeding when brushing teeth, can influence the course of pregnancy and/or the health of the baby at birth?
   1. Yes
   2. No
6. Have you ever heard of a possible relationship between oral health and pregnancy?
   1. Yes
   2. No
7. If the answer is ‘yes’, where did you hear this? (more than one answer possible)
   1. I read it in a book/journal/online resources
   2. My gynecologist told me about it
   3. My dentist/dental hygienist told me about it
   4. I heard it from friends/colleagues/relatives
   5. I experienced this during pregnancy
8. Did you receive advice on your oral health during pregnancy or on the future health of your child?
   1. Yes
   2. No
9. During pregnancy, who give you advice on your oral health or that future of the baby?
   1. General practitioner
   2. Gynecologist
   3. Obstetrician
   4. Dentist/dental hygienist
   5. None
   6. Other (specify)

**Part 3. Baby‘s oral health**

1. Are primary teeth important?
   1. Yes
   2. No
   3. I don’t know
2. Do you know the causes of caries?
   1. Yes
   2. No
   3. I don’t know
3. Is good oral health of the baby linked to good general health?
   1. Yes
   2. No
   3. I don’t know
4. The best option for treating a carious primary tooth is?
   1. Extraction
   2. Restoration
   3. Drug (antibiotic/pain relief drug)
   4. I don’t know
5. At what age should the child go to the dentist?
   1. During the first year of age
   2. Before 3 years of age
   3. Before 6 years of age
   4. After 6 years of age
   5. Only in case of oral problems
   6. I don’t know
6. A balanced diet is essential for a child's growth and oral health.
   1. I agree
   2. I don’t agree
   3. I don’t know
7. Bottle-feeding or breastfeeding overnight can cause caries
   1. I agree
   2. I don’t agree
   3. I don’t know
8. Prolonged and frequent bottle feeding or breastfeeding, especially after the child's first year of life, can cause caries.
9. I agree
10. I don’t agree
11. I don’t know

S2. Content Validity Index and Content Validity Ratio

|  | Item |  | Category |  | Subcategory | VCI(R) | VCI(C) | VCI | CVR |
| --- | --- | --- | --- | --- | --- | --- | --- | --- | --- |
| 1 | Please indicate which of the following age groups you fall into: |  |  |  |  |  |  |  |  |
|  |  | 1 | < 25 years of age |  |  | 1.00 | 1.00 | 1.00 | 1.00 |
|  |  | 2 | 25-30 years of age |  |  | 1.00 | 1.00 | 1.00 | 1.00 |
|  |  | 3 | 31-35 years of age |  |  | 1.00 | 1.00 | 1.00 | 1.00 |
|  |  | 4 | >40 years of age |  |  | 1.00 | 1.00 | 1.00 | 1.00 |
| 2 | Your nationality is: |  |  |  |  |  |  |  |  |
|  |  | 5 | Italian |  |  | 1.00 | 1.00 | 1.00 | 1.00 |
|  |  | 6 | other nationality |  |  | 1.00 | 1.00 | 1.00 | 1.00 |
| 3 | Your occupation is: |  |  |  |  |  |  |  |  |
|  |  | 7 | unemployed |  |  | 1.00 | 1.00 | 1.00 | 1.00 |
|  |  | 8 | housewife |  |  | 1.00 | 1.00 | 1.00 | 1.00 |
|  |  | 9 | occasional job |  |  | 1.00 | 1.00 | 1.00 | 1.00 |
|  |  | 10 | employee |  |  | 1.00 | 1.00 | 1.00 | 1.00 |
|  |  | 11 | self-employed |  |  | 1.00 | 1.00 | 1.00 | 1.00 |
| 4 | How many children do you have? |  |  |  |  |  |  |  |  |
|  |  | 12 | 1 |  |  | 1.00 | 1.00 | 1.00 | 1.00 |
|  |  | 13 | 2 |  |  | 1.00 | 1.00 | 1.00 | 1.00 |
|  |  | 14 | 3 |  |  | 1.00 | 1.00 | 1.00 | 1.00 |
|  |  | 15 | 4 |  |  | 1.00 | 1.00 | 1.00 | 1.00 |
|  |  | 16 | >4 |  |  | 1.00 | 1.00 | 1.00 | 1.00 |
| 5 | How old is your younger child? |  |  |  |  |  |  |  |  |
|  |  | 17 | 0-6 months |  |  | 1.00 | 1.00 | 1.00 | 0.80 |
|  |  | 18 | 7-12 months |  |  | 1.00 | 1.00 | 1.00 | 0.80 |
|  |  | 19 | 13-24 months |  |  | 1.00 | 1.00 | 1.00 | 0.80 |
|  |  | 20 | 25-36 months |  |  | 1.00 | 1.00 | 1.00 | 0.80 |
| 6 | Did you experience any problems with your teeth or gums during pregnancy? |  |  |  |  |  |  |  |  |
|  |  | 21 | Yes |  |  | 1.00 | 1.00 | 1.00 | 1.00 |
|  |  | 22 | No |  |  | 1.00 | 1.00 | 1.00 | 1.00 |
| 7 | Did you regularly visit the dentist/dental hygienist before your pregnancy? |  |  |  |  |  |  |  |  |
|  |  | 23 | Yes |  |  | 1.00 | 1.00 | 1.00 | 1.00 |
|  |  | 24 | No |  |  | 1.00 | 1.00 | 1.00 | 1.00 |
| 8 | Did you visit the dentist/dental hygienist during pregnancy? |  |  |  |  |  |  |  |  |
|  |  | 25 | Yes |  |  | 1.00 | 1.00 | 1.00 | 1.00 |
|  |  | 26 | No |  |  | 1.00 | 1.00 | 1.00 | 1.00 |
|  |  |  |  | 1 | If the answer is ‘no’, give one closest reason on why you didn’t go to the dentist |  |  |  |  |
|  |  |  |  | 2 | Because I thought my gums would soon recover | 0.40 | 0.60 | 0.50 | 0.80 |
|  |  |  |  | 3 | Because I knew from my previous pregnancy that mouth problems can be pregnancy-related | 0.60 | 1.00 | 0.80 | 0.80 |
|  |  |  |  | 4 | Because the dentist could have used a local anesthetic or prescribed me antibiotics without consulting my gynecologist | 1.00 | 1.00 | 1.00 | 1.00 |
|  |  |  |  | 5 | Because the dentist/dental hygienist could not have done any treatment during pregnancy | 1.00 | 1.00 | 1.00 | 1.00 |
|  |  |  |  | 6 | Because I had no oral problem during pregnancy | 1.00 | 1.00 | 1.00 | 1.00 |
|  |  |  |  | 7 | No specific reason | 1.00 | 1.00 | 1.00 | 1.00 |
|  |  |  |  | 8 | Other reason | 1.00 | 1.00 | 1.00 | 1.00 |
| 9 | Do you think that gum problems, such as bleeding when brushing teeth, can influence the course of pregnancy and/or the health of the baby at birth? |  |  |  |  |  |  |  |  |
|  |  | 27 | Yes |  |  | 1.00 | 1.00 | 1.00 | 1.00 |
|  |  | 28 | No |  |  | 1.00 | 1.00 | 1.00 | 1.00 |
| 10 | Have you ever heard of a possible relationship between oral health and pregnancy? |  |  |  |  |  |  |  |  |
|  |  | 29 | Yes |  |  | 1.00 | 1.00 | 1.00 | 1.00 |
|  |  | 30 | No |  |  | 1.00 | 1.00 | 1.00 | 1.00 |
|  |  |  |  | 9 | If the answer is ‘yes’, where did you hear this? (more than one answer possible) |  |  |  |  |
|  |  |  |  | 10 | I read it in a book/journal/online resources | 1.00 | 1.00 | 1.00 | 1.00 |
|  |  |  |  | 11 | My gynecologist told me about it | 1.00 | 1.00 | 1.00 | 1.00 |
|  |  |  |  | 12 | My dentist/dental hygienist told me about it | 1.00 | 1.00 | 1.00 | 1.00 |
|  |  |  |  | 13 | I heard it from friends/colleagues/relatives | 1.00 | 1.00 | 1.00 | 1.00 |
|  |  |  |  | 14 | I experienced this during pregnancy | 0.60 | 1.00 | 0.80 | 0.80 |
| 11 | Did you receive advice on your oral health during pregnancy or on the future health of your child? |  |  |  |  |  |  |  |  |
|  |  | 31 | Yes |  |  | 1.00 | 1.00 | 1.00 | 1.00 |
|  |  | 32 | No |  |  | 1.00 | 1.00 | 1.00 | 1.00 |
| 12 | During pregnancy, who give you advice on your oral health or that future of the baby? |  |  |  |  |  |  |  |  |
|  |  | 33 | General practitioner |  |  | 1.00 | 1.00 | 1.00 | 1.00 |
|  |  | 34 | Gynecologist |  |  | 1.00 | 1.00 | 1.00 | 1.00 |
|  |  | 35 | Obstetrician |  |  | 1.00 | 1.00 | 1.00 | 1.00 |
|  |  | 36 | Dentist/dental hygienist |  |  | 1.00 | 1.00 | 1.00 | 1.00 |
|  |  | 37 | None |  |  | 1.00 | 1.00 | 1.00 | 1.00 |
|  |  | 38 | Other (specify) |  |  | 1.00 | 1.00 | 1.00 | 1.00 |
| 13 | Are primary teeth important? |  |  |  |  |  |  |  |  |
|  |  | 39 | Yes |  |  | 1.00 | 1.00 | 1.00 | 1.00 |
|  |  | 40 | No |  |  | 1.00 | 1.00 | 1.00 | 1.00 |
|  |  | 41 | I don’t know |  |  | 1.00 | 1.00 | 1.00 | 1.00 |
| 14 | Do you know the causes of caries? |  |  |  |  |  |  |  |  |
|  |  | 42 | Yes |  |  | 1.00 | 1.00 | 1.00 | 1.00 |
|  |  | 43 | No |  |  | 1.00 | 1.00 | 1.00 | 1.00 |
|  |  | 44 | I don’t know |  |  | 0.40 | 1.00 | 0.70 | 0.80 |
| 15 | Is good oral health of the baby linked to good general health? |  |  |  |  |  |  |  |  |
|  |  | 45 | Yes |  |  | 1.00 | 1.00 | 1.00 | 1.00 |
|  |  | 46 | No |  |  | 1.00 | 1.00 | 1.00 | 1.00 |
|  |  | 47 | I don’t know |  |  | 1.00 | 1.00 | 1.00 | 1.00 |
| 16 | The best option for treating a carious primary tooth is? |  |  |  |  |  |  |  |  |
|  |  | 48 | Extraction |  |  | 1.00 | 1.00 | 1.00 | 1.00 |
|  |  | 49 | Restoration |  |  | 1.00 | 1.00 | 1.00 | 1.00 |
|  |  | 50 | Drug (antibiotic/pain relief drug) |  |  | 1.00 | 1.00 | 1.00 | 1.00 |
|  |  | 51 | I don’t know |  |  | 1.00 | 1.00 | 1.00 | 1.00 |
| 17 | At what age should the child go to the dentist? |  |  |  |  |  |  |  |  |
|  |  | 52 | During the first year of age |  |  | 1.00 | 1.00 | 1.00 | 1.00 |
|  |  | 53 | Before 3 years of age |  |  | 1.00 | 1.00 | 1.00 | 1.00 |
|  |  | 54 | Before 6 years of age |  |  | 1.00 | 1.00 | 1.00 | 1.00 |
|  |  | 55 | After 6 years of age |  |  | 1.00 | 1.00 | 1.00 | 1.00 |
|  |  | 56 | Only in case of oral problems |  |  | 1.00 | 1.00 | 1.00 | 1.00 |
|  |  | 57 | I don’t know |  |  | 1.00 | 1.00 | 1.00 | 1.00 |
| 18 | A balanced diet is essential for a child's growth and oral health. |  |  |  |  |  |  |  |  |
|  |  | 58 | I agree |  |  | 1.00 | 1.00 | 1.00 | 1.00 |
|  |  | 59 | I don’t agree |  |  | 1.00 | 1.00 | 1.00 | 1.00 |
|  |  | 60 | I don’t know |  |  | 1.00 | 1.00 | 1.00 | 1.00 |
| 19 | Bottle-feeding or breastfeeding overnight can cause caries. |  |  |  |  |  |  |  |  |
|  |  | 61 | I agree |  |  | 1.00 | 1.00 | 1.00 | 1.00 |
|  |  | 62 | I don’t agree |  |  | 1.00 | 1.00 | 1.00 | 1.00 |
|  |  | 63 | I don’t know |  |  | 1.00 | 1.00 | 1.00 | 1.00 |
| 20 | Prolonged and frequent bottle feeding or breastfeeding, especially after the child's first year of life, can cause caries. |  |  |  |  |  |  |  |  |
|  |  | 64 | I agree |  |  | 1.00 | 1.00 | 1.00 | 1.00 |
|  |  | 65 | I don’t agree |  |  | 1.00 | 1.00 | 1.00 | 1.00 |
|  |  | 66 | I don’t know |  |  | 1.00 | 1.00 | 1.00 | 1.00 |
|  | **Overall** |  |  |  |  | **0.97** | **0.99** | **0.98** | **0.98** |
